# Exome sequencing and prenatal skeletal abnormalities: comprehensive review and meta-analysis and way forward

**DOI:** 10.3389/fgene.2025.1502538

**Published:** 2025-06-11

**Authors:** Mengting Jiang, Bin Zhang, Jing Wang, Cui Wei, Xiuzhen Mao, Bin Yu

**Affiliations:** ^1^ Department of Medical Genetics, Changzhou Maternal and Child Healthcare Hospital, Changzhou Medical Center of Nanjing Medical University, Changzhou, Jiangsu, China; ^2^ The Affiliated Suqian First People’s Hospital of Nanjing Medical University, Suqian First People's Hospital, Suqian, Jiangsu, China

**Keywords:** exome sequencing, skeletal abnormalities, prenatal diagnosis, karyotyping, chromosomal microarray analysis

## Abstract

**Objective:**

To assess the detection rate of exome sequencing (ES) in fetuses diagnosed as skeletal abnormalities (SKA) with normal karyotype or chromosomal microarray analysis (CMA) results.

**Methods:**

We conducted electronic searches in four databases, focusing on studies involving ES in fetuses with SKA. Additional detection rate of ES compared to karyotype/CMA was calculated, followed by a meta-analysis. Subgroup analyses explored the influence of fetal phenotype on diagnostic outcomes.

**Results:**

From 2,393 studies, 21 reports covering 476 fetuses were analyzed. Key findings include: (1) an additional detection rate of ES of 63.2% (Risk Difference (RD), 0.68 [95% CI, 0.60–0.76], p < 0.00001); (2) identification of 76 genes across 304 types of variants, with *FGFR3*, *COL1A1*, *COL1A2*, and *COL2A1* being prevalent; (3) lower detection rates in fetuses with isolated short long bones compared to non-isolated conditions, though not significantly different (p = 0.35); (4) higher detection rates in subgroups with abnormal ossification, small chest, suspected long bone fractures or angulations, and skull abnormalities.

**Conclusion:**

The meta-analysis indicates that genetic variation significantly contributes to fetal SKA, primarily due to single-gene variants. Consequently, ES should be used in the prenatal diagnosis of SKA fetuses in clinical practice.

## 1 Introduction

Fetal skeletal abnormalities (SKA) is a prevalent structural malformation, occurring in approximately 5 per 1,000 fetuses (Schramm and Mommsen, 2018). This congenital disease impacts the composition and structure of bone and cartilage tissue. Clinical manifestations of SKA include various abnormalities in skeletal tissue growth and development, such as short stature, joint malposition, cranial and limb deformities, abnormal spinal curvature, and changes in bone mineral density. Additionally, this condition may co-occur with malformations in other systems and organs (Schramm and Mommsen, 2018; Krakow, 2015; Milks et al., 2017). In severe cases, abnormal skeletal development in fetuses can lead to fetal death. Survivors may face disabilities due to skeletal malformations, and in some instances, various degrees of intellectual disability, severely affecting patient quality of life (Schramm and Mommsen, 2018). Current research indicates that genetic factors, such as chromosomal number and structural abnormalities, chromosomal copy number variations, and single-gene variants, are considered to be the main factors leading to fetal skeletal abnormalities (Krakow, 2015; Kucinska-Chahwan et al., 2022). The impact of SKA on fetal health is significant, being a major cause of birth defects. Therefore, early detection through prenatal screening and diagnosis is crucial for timely clinical intervention. Currently, ultrasound examination is the most effective prenatal diagnostic tool for SKA. However, its efficacy is influenced by several factors, including gestational age, fetal position, maternal abdominal wall conditions, the types of SKA, amniotic fluid volume, and variations in sonographer techniques and experience. These factors contribute to the limitations of ultrasound in the detection of SKA fetuses (Best et al., 2018). In clinical practice, genetic testing is often conducted on fetuses diagnosed with SKA via ultrasound. Traditional genetic diagnosis methods include karyotyping and chromosomal microarray analysis (CMA) of amniotic fluid exfoliated cells (Wapner et al., 2012; Callaway et al., 2013). Most SKA fetuses are monogenic genetic diseases (Best et al., 2018), so the current first-line prenatal diagnosis techniques, karyotyping and CMA, are not fully suitable for SKA fetuses. Consequently, prenatal screening and diagnosis that rely solely on ultrasound combined with chromosome analysis may not be entirely sufficient for diagnosing the genetic etiology of fetal SKA.

Exome Sequencing (ES) encompasses 1%–2% of the genome, yet includes about 85% of known disease-causing genetic variants. Two prospective studies have shown disease detection rates of 24% and 15.4% in SKA fetuses when assessed on a large scale using ES (Tournis and Dede, 2018; Lord et al., 2019). Additionally, a meta-analysis of 66 studies and 72 reports underscored the significant value of ES in prenatal detection. This study found that for fetuses with structural abnormalities, the detection rate using ES was 31% higher compared to CMA or karyotyping. Notably, the detection rate for SKA was the highest at 53% (Mellis et al., 2022).

However, due to the rarity of SKA fetuses, most studies evaluating the additional detection rate of prenatal ES in these cases have been limited to small sample sizes. Moreover, the complexity of SKA types contributes to a lack of data accumulation and evidence guiding the selection of prenatal diagnosis techniques and genetic counselling. In our study, we merged various studies on the application of ES in SKA fetuses with normal karyotypes or CMA results to form a larger cohort. We then conducted a meta-analysis to explore the additional detection value of ES in SKA fetuses with normal karyotypes or CMA.

## 2 Materials and methods

### 2.1 Protocol

We devised a systematic review protocol in line with PRISMA guidance (Moher et al., 2009; Page et al., 2021). Study authors agreed the protocol prior to conducting the searches. Any required small amendments were made with the consensus of all authors.

### 2.2 Data sources and search strategies

#### 2.2.1 Literature source

Searches were conducted in PubMed, EMBASE, Web of Science, and the Cochrane Library for articles published up to May 2023.

#### 2.2.2 Search strategy

This involved using a combination of subject terms and keywords. 1) For English subject terms: ‘skeletal dysplasia’ was represented by ‘skeletal abnormalities’, ‘SD’, ‘SKA’, ‘SDs’, and ‘skeletal dysplasia’s’. Similarly, ‘whole exome sequencing’ and ‘WES‘ were used to refer to ‘exome sequencing’. 2) Additionally, references from identified literature were reviewed to locate other relevant studies. 3) Finally, two researchers independently conducted and evaluated the literature search.

### 2.3 Eligibility criteria

#### 2.3.1 Inclusion criteria

1) Ultrasound suggested fetal skeletal abnormalities, including SKA fetuses, SKA fetuses with other skeletal or non-skeletal abnormalities. 2) Prospective or retrospective cohort studies. 3) Prenatal ES including Whole Exome Sequencing (WES), Targeted Exome Sequencing (TES), or SKA panel, for diagnosing SKA fetuses. This encompasses studies based on prenatal phenotypes with ES testing completed post-delivery. 4) Cases classified as pathogenic or likely pathogenic variats according to the ACMG (American College of Medical Genetics and Genomics) guidelines and identified as the cause of the fetal ultrasound phenotype, were included in the study. However, cases with only one pathogenic or likely pathogenic variant of an autosomal recessive inheritance, without a second variant, as well as those with Variants of Uncertain Significance (VOUS), were not considered diagnostic cases. Cases where karyotype or CMA results were negative or no diagnostic, and where numerical, structural, and copy number abnormalities (CNVs) of chromosomes were ruled out. 5) Studies that described specific prenatal ultrasound phenotypes. 6) Studies that provide raw data. 7) Full-text reports available in the English language.

#### 2.3.2 Exclusion criteria

1) Literature types such as reviews, case reports, reader letters, animal studies, expert opinions, conference papers, etc.. 2) Studies that did not use Exome Sequencing. 3) Studies lacking a specific subgroup for SKA. 4) Studies where data could not be extracted.

### 2.4 Study selection

Following the removal of duplicate content, two investigators independently examined titles and abstracts. For abstracts deemed potentially relevant, full texts were scrutinized based on predetermined inclusion and exclusion criteria. Any disagreements between the investigators were resolved through discussion. To prevent data duplication, only studies with the largest or most recent sample sizes were included.

### 2.5 Data extraction

Data were independently extracted by two investigators, including study setting, sample size, ES method, number of fetuses diagnosed, pregnancy outcomes, and others. If these details were not specified in the literature, the authors were contacted via email for clarification.

### 2.6 Quality assessment of included studies

Two researchers independently evaluated the quality of the included studies. For this evaluation, the criteria recommended by the Agency for Healthcare Research and Quality (AHRQ) were utilized.

### 2.7 Data statistics

The incremental diagnostic value of prenatal ES over CMA or karyotype analysis was determined using the 95% confidence interval (CI) from each study. Risk differences were calculated using a random effects model. Statistical analysis was conducted using RevMan 5.3. A statistical difference was considered significant when P < 0.05.

### 2.8 Heterogeneity test

Heterogeneity within the studies was evaluated using the I^2^ statistic in the forest plot. An I^2^ = 0 indicated no heterogeneity. Conversely, a larger I^2^ statistic reflected greater heterogeneity. Typically, an I^2^ > 50% suggested significant heterogeneity. The threshold for significance in this study was set at 0.1. If P > 0.1 and I^2^ was <50%, it was interpreted as a lack of heterogeneity among the studies. If these criteria were not met, heterogeneity among the studies was presumed.

### 2.9 Assessment of literature publication bias

The analysis results were represented by a Funnel plot, a scatter plot with the size of the effect on the abscissa (horizontal axis) and the sample size on the ordinate (vertical axis). In the absence of publication bias among the included studies, this plot typically exhibits a symmetrical funnel shape.

## 3 Results

### 3.1 Data and materials

**FIGURE 1:**
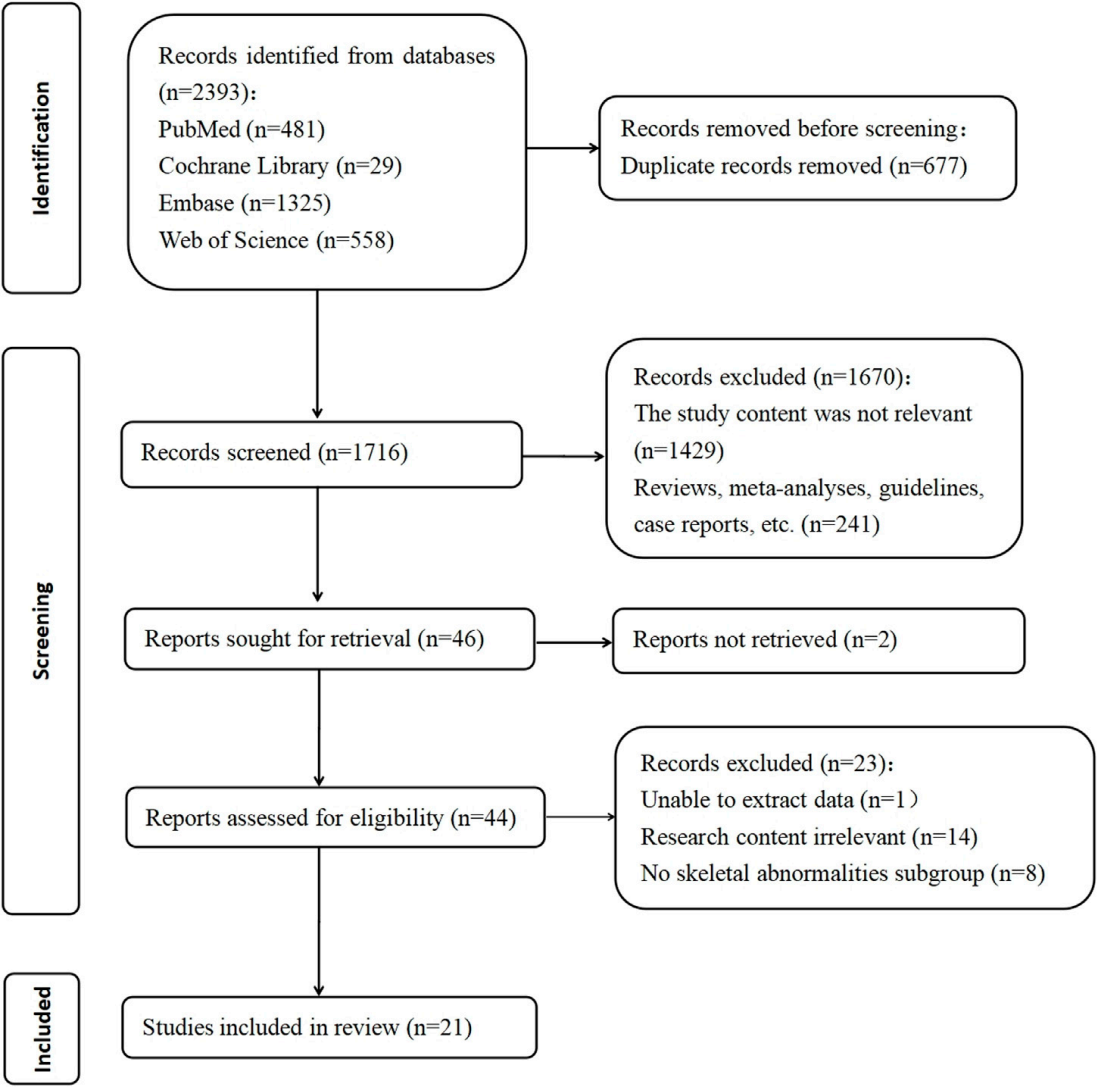
PRISMA flow diagram showing study screening and selection.

A total of 2,393 manuscripts were retrieved using the specified database. Following the initial exclusion based on title and abstract reading, 46 pieces remained. After a full-text review, 25 pieces were further excluded due to reasons such as the inability to extract relevant data, irrelevance to the research topic, or the absence of the full text. Ultimately, 21 manuscripts that met the inclusion criteria were analyzed, and are listed in Table 1. The process of literature selection is depicted in Figure 1. The quality of these 21 articles was independently assessed based on the 11 items recommended by AHRQ (Supplementary Table 1).

**TABLE 1:** Summary of studies included in this study.

Studies	Region	Year	Inclusion criteria	Sequencing methods	Number of ES cases	Overall detection rate
Cao et al. (2022)	China	2022	SKA on US	Solo or Trio WES	8	87.5% (7/8)
Zhang et al. (2021b)	China	2021	SKA on US, lethality was assessed for fetal FL/AC<0.16	Trio ES or Trio SKA panel	27	70.4% (19/27)
Zhang et al. (2021a)	China	2021	SKA on US	Solo or Trio WES	55	63.6% (35/55)
Yang et al. (2019)	China	2019	SKA on US	Trio WES	8	75.0% (6/8)
Yang et al. (2022)	China	2022	SKA on US	Trio WES	5	100.0% (5/5)
Huang et al. (2023)	China	2023	SKA on US, FL and/or other long bones<−2SD with/without other abnormalities	Trio ES	94	45.7% (38/94)
Kucinska-Chahwan et al. (2022)	Poland	2022	SKA on US	WES	26	69.2% (16/26)
Bai et al. (2022)	China	2022	SKA on US	Trio ES or Trio SKA panel	48	79.2% (38/48)
Peng et al. (2021)	China	2021	SKA on US, FL < −2SD or FL < 5th centile+/−various deformities, finger/toe deformities, missing fingers/toes, and/or absence of upper/lower limbs or other skeletal anomalies	Trio WES	38	63.2% (24/38)
Tang et al. (2021)	China	2021	SKA on US	Trio WES	15	(66.7% (10/15)
Deden et al. (2020)	Netherlands	2019	SKA on US	Trio rWES	19	(57.9% (11/19)
Chandler et al. (2018)	Britain	2018	SKA on US	Trio TES	16	81.3% (13/16)
Han et al. (2020)	China	2020	SKA on US, long bones<5th centile+/−bowing or fracturing, hypo mineralization, hydrops, thoracic hypoplasia or other malformations	Trio ES	26	88.5% (23/26)
Tang et al. (2020)	China	2020	SKA on US, FL < 5th centile	Trio WES	8	75.0% (6/8)
Liu et al. (2019)	China	2019	Local skeletal deformity and systemic general skeletal dysplasia	Trio TES	28	57.1% (16/28)
Tolusso et al. (2021)	United States	2021	SKA on US	ES	14	57.1% (8/14)
Zhou et al. (2018)	China	2018	SKA on U	Trio SKA panel	12	75.0% (9/12)
Aggarwal et al. (2019)	India	2020	SKA on US	ES	11	54.6% (6/11)
Jelin et al. (2020)	United States	2020	SKA on US	Trio SKA panel	9	77.8% (7/9)
Yadava and Ashkinadze (2018)	United States	2018	SKA on US	Trio WES or Trio SKA panel	4	75.0% (3/4)
Vora et al. (2017)	United States	2017	SKA on US	Trio ES	5	20.0% (1/5)
Total					476	63.2% (301/476)

Note: Trio rWES: Trio-based rapid whole exome sequencing; Trio TES: trio targeted exome sequencing; SKA, skeletal abnormalities; FL: femur length; AC: abdominal circumference; US, ultrasound; ES: exome sequencing; rWES: rapid whole exome sequencing; TES: targeted exome sequencing.

### 3.2 Study characteristics

Among the 21 studies included in this analysis, 13 (encompassing 372 patients) were conducted in China. The meta-analysis included a total of 476 eligible fetuses, with a mean gestational age of 24 weeks, ranging from 12 to 40^+2^ weeks. At the time of drafting, 257 pregnancies had been terminated, while there were 51 live births, 5 stillbirths, 12 neonatal deaths, and 3 ongoing pregnancies. The pregnancy outcomes for the remaining 148 patients were not described. Detailed information on phenotype-genotype correlations and molecular diagnoses can be found in the Supplementary Material under ‘Genes’.

### 3.3 Overall additional monogenic disorder detection rate of ES

Excluding chromosomal abnormalities, the overall rate of abnormal ES testing in fetuses with SKA was 63.2% (Table 1). This result shows a RD of 0.68 with a 95% CI of 0.60–0.76, and p < 0.00001 (Supplementary Figure S1).

Of the 301 cases with positive ES results, 235 cases (78.1%) were autosomal dominant (199 cases *de-novo* vs. 35 cases inherited), while 66 cases (21.9%) were autosomal recessive (11 cases homozygous vs. 45 cases compound heterozygous) (7 cases *de-novo* vs. 49 cases inherited). A further 11 cases either did not perform parental testing, or the results of parental analysis were not mentioned in the studies. In these 301 cases, a total of 76 genes across 304 types of variants were detected. The ten most common genes identified were *FGFR3*, *COL1A1*, *COL1A2*, *COL2A1*, *DYNC2H1*, *ALPL*, *FLNB*, *EBP*, *PPIB*, and *IFITM5*. Their respective frequencies were 31.3%, 14.5%, 10.2%, 8.9%, 3.6%, 2.3%, 1.3%, 1.3%, 1.3%, and 1.0% (Supplementary Figure S2).

### 3.4 Subgroup analysis of monogenic disorder detection rates

To delve deeper into the individual sonographic features of SKA and their respective clinical implications, a subgroup analysis was conducted. This analysis was based on the described sonographic features, with subgroup categorization partially referencing the groupings from the study by Tse et al. (Tse et al., 2023) and the detailed subgroup and results are presented in Table 2 and Supplementary Table 2.

**TABLE 2:** Additional detection rate of ES in the SKA subgroups over karyotype or CMA.

Phenotypes	Detection rate of ES	RD [95%CI]	Hot spot mutation gene
1 SKA	63.2% (301/476)	0.68 [0.60, 0.76]	FGFR3, COL1A1, COL1A2, COL2A1
1.1 Isolated SKA	68.6% (203/296)	0.71 [0.64, 0.79]	FGFR3, COL1A1, COL1A2, COL2A1
1.2 SKA combined with other abnormalities	54.4% (98/180)	0.59 [0.49, 0.70]	FGFR3、COL1A1、COL1A2、COL2A1
2 Short long bones	67.0% (250/373)	0.72 [0.64, 0.80]	FGFR3, COL1A1, COL1A2, COL2A1
2.1 Isolated short long bones	61.9% (52/84)	0.63 [0.41, 0.86]	FGFR3, COL1A1, COL1A2, COL2A1
2.2 Non-isolated short long bones	68.5% (198/289)	0.75 [0.66, 0.85]	FGFR3, COL1A1, COL1A2, COL2A1
2.2.1 Short long bones only combined with non-skeletal abnormalities	42.1% (24/57)	0.61 [0.38, 0.84]	FGFR3, COL2A1
2.2.2 Short long bones only combined with other skeletal abnormalities	79.1% (129/163)	0.82 [0.76, 0.89]	FGFR3, COL1A1, COL1A2, COL2A1
2.2.3 Short long bones combined with both other skeletal abnormalities and non-skeletal abnormalities	65.2% (45/69)	0.69 [0.57, 0.80]	FGFR3, COL2A1, COL1A1, COL1A2
3 Short long bones with specific descriptions of long bone length	30.3% (9/30)	0.28 [0.11, 0.45]	FGFR3
3.1 <-2SD	22.2% (2/9)	0.22 [-0.09, 0.52]	COL1A2, COL2A1
3.2 <-4SD	50.0% (4/8)	0.35 [-0.15, 0.84]	FGFR3, CUL7
3.3 <-3SD	23.1% (3/13)	0.22 [-0.03, 0.48]	FGFR3, RARB
4 Abnormal curvature of long bones	75.8% (91/120)	0.79 [0.70, 0.88]	FGFR3, COL1A1, COL1A2, ALPL
4.1 Isolated curvature abnormalities of long bones	100.0% (3/3)	1.00 [0.51, 1.49]	COL1A1, COL1A2, DYNC2H1
4.2 Long bone curvature abnormalities associated with short long bones only	82.6% (38/46)	0.86 [0.74, 0.99]	COL1A1, COL1A2, FGFR3
4.3 Long bone curvature abnormalities combined with other skeletal abnormalities and non-skeletal abnormalities	70.4% (50/71)	0.73 [0.58, 0.88]	FGFR3, COL1A1, COL1A2
5 Subgroup phenotype			
5.1 Suspected fractures or angulated long bones	78.1% (25/32)	0.79 [0.62, 0.96]	COL1A1, COL1A2, PPIB
5.2 Reduced or abnormal ossification	85.0% (17/20)	0.85 [0.67, 1.04]	COL1A1, COL2A1, COL1A2
5.3 Absence of long bones, absence or abnormality of the bones of the fingers or toes	31.3% (15/48)	0.28 [0.14, 0.41]	DYNC2H1, TP63
5.4 Abnormal joint posture including talus, contracture and foot varus	48.5% (33/68)	0.50 [0.34, 0.65]	COL2A1, COL1A1, COL1A2
5.5 Abnormalities of the skull	77.8% (35/45)	0.76 [0.58, 0.93]	FGFR3, COL1A1, COL1A2
5.6 Facial abnormalities including retrognathia/micrognathia, nasal bone loss and facial flatness	55.9% (19/34)	0.56 [0.38, 0.73]	COL2A1, FGFR3, COL1A1, NEB
5.7 Small breasts including bell-shaped breasts	81.5% (66/81)	0.87 [0.78, 0.96]	FGFR3, COL1A1, COL2A1, DYNC2H1
5.8 Spinal abnormalities including scoliosis	55.3% (21/38)	0.47 [0.26, 0.69]	FGFR3, COL2A1
5.9 Edema including cystic edema, subcutaneous edema and pleural effusion	52.2% (12/23)	0.56 [0.26, 0.87]	COL2A1, COL1A1
5.10 Multiple system anomaly	54.9% (45/82)	0.58 [0.43, 0.74]	FGFR3, COL2A1

Among the 476 SKA fetuses included in the study, the detection rate of isolated SKA fetuses was 68.6% (203/296), and the detection rate of non-isolated SKA fetuses was 54.4% (98/180), but the two groups did not achieve statistical significance (p = 0.07). Our study revealed that the detection rate of fetuses with isolated short long bones (52/84, 61.9%) was lower compared to those with non-isolated short long bones (198/289, 68.5%). However, this difference was not statistically significant (p = 0.35). Among the 84 fetuses with isolated short long bones, 30 cases had a detailed description of the length of the long bones shortening. Among these, the femur length of 9 cases was less than −2 standard deviations (SD) of the fetus of the same gestational age, 13 were less than –3SD, and eight were less than –4SD. The detection rates of ES in these subgroups were 22.2%, 23.1%, and 50.0%, respectively. When comparing fetuses with short bones less than –2SD to those with short bones below –4SD, the latter were more likely to be detected genetic abnormality with ES (22.2% for –2SD versus 50.0% for –4SD), but this difference did not reach statistical significance (p = 0.66). In the 9 fetuses with long bones less than –2SD, two cases were still diagnosed by ES. The detection rate of short long bone only combined with other skeletal abnormalities (129/163, 79.1%) was significantly higher than that of short long bone combined with both other skeletal abnormalities and non-skeletal abnormalities (65.2%, 45/69) and p value was 0.04.

Among the 120 fetuses with abnormal curvature of long bones, 111 cases also presented with varying degrees of short long bones. In the three cases with isolated long bone curvature abnormalities, all underwent ES tests which were positive. Two of these cases had only a slight curvature of the long bones, with variant genes identified as *COL1A1*, *COL1A2*, and *DYNC2H1*, respectively. In the 46 cases where abnormal curvature of long bones was combined with short long bones, the detection rate for ES abnormalities was as high as 82.6% (38/46).

Subgroups with higher additional ES detection rates included abnormal ossification (85.0%), small thorax (81.5%), suspected fractures or angulations of long bones (78.1%), and skull abnormalities (77.8%). Conversely, fetuses with absent long bones and abnormal joint posture exhibited relatively lower extra detection rates of 31.3% and 48.5%, respectively, when undergoing ES tests.

The most frequently mutated genes in the long bone loss subgroup were *DYNC2H1* and *TP63*. In other subgroups, the most common variants were found in *FGFR3*, *COL1A1*, *COL1A2*, and *COL2A1*.

Heat map analysis of the 10 most common variant genes related to fetal SKA in the meta-analysis results revealed that *FGFR3* had the highest frequency in the subgroup of short long bones with other skeletal abnormalities. In isolated short long bones, *FGFR3*, and in the subgroup of short long bones with other skeletal abnormalities, *COL1A1*, had the second highest frequency. *FGFR3* was also the third most frequent in the small thorax subgroup (Supplementary Figure S3).

### 3.5 Heterogeneity and publication bias analysis

Given the high level of heterogeneity observed, a random-effects model was employed, although heterogeneity remained relatively substantial. The funnel plot used for the publication bias test displayed significant asymmetry, suggesting the potential presence of publication bias (Supplementary Figure S4).

## 4 Discussion

### 4.1 Diagnostic rate

Our review underscores the significance of prenatal ES in fetuses with SKA. The findings reveal that the overall abnormal detection rate of ES compared to karyotyping or CMA was 63.2%. In cases of suspected fetal SKA, the detection rate for CMA testing ranged only between 1.7% and 7.9% (de Wit et al., 2014; Hui et al., 2021). This lower rate is attributed to the fact that SKA is primarily a monogenic disorder, which CMA cannot detect. Pure skeletal dysplasia or classical skeletal dysplasia is a Mendelian monogenic disease (Krakow, 2015), and the detection rate of karyotype or CMA may be lower. Consequently, our research advocates for the routine use of ES in prenatal testing for suspected cases of SKA.

Our study indicated that the detection rate in fetuses with isolated short long bones (61.9%) was slightly lower compared to those with non-isolated short long bones (68.5%), yet this difference was not statistically significant (p = 0.35). This finding aligns with the conclusions of Tse et al.'s study (Tse et al., 2023). We also observed a gradual increase in ES detection rates in fetuses with long bone lengths less than –2SD, –3SD, and –4SD (22.2%, 23.1%, and 50.0%, respectively). In practice, obstetricians frequently encounter pregnancies with fetal short long bones, but few undergo invasive prenatal examinations. However, our data suggest that ES is also crucial for fetuses with mildly shortened long bones.

This study also found that abnormal curvature of long bones often co-occurs with short long bones. In our subgroup analysis, 92.5% (111/120) of cases with abnormal long bone curvature also had varying degrees of short long bones. Among 46 cases with both abnormal curvature and short long bones, the detection rate was high at 82.6%. In three cases of isolated long bone curvature abnormalities, all ES tests were positive, including two with only mild curvature. Thus, fetuses with abnormal curvature of long bones are more likely to carry pathogenic genes related to bone conditions. Clinicians should therefore give considerable attention to such fetal abnormalities in clinical practice.

The detection rate of short long bone only combined with other skeletal abnormalities (129/163, 79.1%) was higher than that of short long bone combined with both other skeletal abnormalities and non-skeletal abnormalities (45/69, 65.2%), and the difference was statistically significant (p = 0.04). Therefore, skeletal system malformations combined with non-skeletal system malformations do not necessarily have higher SKA-related pathogenic genes, because some syndrome diseases may also cause skeletal malformations.

Moreover, the study revealed that specific SKA subgroup characteristics might offer additional detection benefits. The subgroups with abnormal ossification, small chests, suspected fractures or angulations of long bones, and skull abnormalities showed relatively high ES detection rates, being 85.0%, 81.5%, 78.1%, and 77.8%, respectively. Therefore, these features should be meticulously assessed during prenatal ultrasounds, as their identification can inform decisions on whether ES should be pursued.

### 4.2 Common variant genes

Nosology of genetic skeletal disorders have identified 771 types of hereditary bone diseases, divided into 41 groups involving 552 genes (Unger et al., 2023). Our meta-analysis revealed that *FGFR3*, *COL1A1*, *COL1A2*, and *COL2A1* are the most frequently mutated genes in SKA fetuses, accounting for 31.3%, 14.5%, 10.2%, and 8.9% of cases, respectively. In subgroup analyses, *DYNC2H1* and *TP63* emerged as prominent variant genes in the long bone loss subgroup, while *FGFR3*, *COL1A1*, *COL1A2*, and *COL2A1* were prevalent in other subgroups. Thus, a gene panel can be utilized in areas with limited resources when typical sonographic features are identified prenatally.

Our study underscores that pathogenic variants in the Fibroblast Growth Factor Receptor 3 (*FGFR3*) and collagen genes are the leading genetic causes of SKA. *FGFR3*, one of four transmembrane tyrosine kinases, serves as a high-affinity receptor for various fibroblast growth factors and plays a crucial role in bone development (Ornitz and Marie, 2015). Pathogenic *FGFR3* variants cause achondroplasia, an autosomal dominant skeletal disorder with an incidence of 2–3 cases per 100,000 people (Waller et al., 2008).

Research indicates that 90% of osteogenesis imperfecta (OI) cases result from pathogenic variants in the *COL1A1* or *COL1A2* genes, which encode the α1 and α2 chains of type I collagen, respectively. These variants affect collagen quantity or structure, with glycine substitutions in the helical domain’s Gly-X-Y triplet being the most common cause of OI (Augusciak-Duma et al., 2018; Zhytnik et al., 2017; Shi et al., 2019; Marini et al., 2017; Petrovski et al., 2019). *COL2A1*, coding for type II collagen, is involved in the regulation of intramembranous and endochondral osteogenesis. Heterozygous variants in *COL2A1* are frequently associated with a range of dwarfism and skeletal dysmorphic disorders (Zhang et al., 2020).

### 4.3 Impact of prenatal exome sequencing on clinical management decisions

ES will play a crucial role in clinical management and parental decision-making. In our study, pathogenic/likely pathogenic variants were detected in 301 of 476 cases, and 290 cases were tested for trio ES, of which 206 cases (68.4%) were *de novo* variants and 84 cases (27.9%) were inherited variants from their parents. 21.9% of the cases were autosomal recessive inheritance with a high risk of recurrence, indicating that trio ES is beneficial for data interpretation and genetic counselling. The remaining 11 cases either did not undergo parental testing or did not mention parental analysis results in the study. The cases without trio ES testing cannot provide a reference for clinicians to provide patients with more comprehensive genetic counseling or provide comprehensive guidance for patients in their next pregnancy.

A significant challenge for obstetricians lies in counselling couples where no genetic cause is identified. The most complex cases are not those with clear, fatal features of SKA, but rather those with mild to moderate features and a normal CMA. In these instances, ES can provide additional insights into the etiology, assisting physicians in offering more informed genetic counselling.

### 4.4 Strengths and limitations

This systematic review and meta-analysis represents a detailed and comprehensive examination of fetal SKA, incorporating 21 studies from four databases. Given the rarity of SKA, most studies have small sample sizes. This review systematically amalgamates these studies to derive an overall diagnostic yield, applying stringent criteria to all studies and excluding all VOUS.

The primary limitation of our review is the high heterogeneity among the included studies, which impacts the accuracy of our comparisons. Even when analyzing the effect of case selection criteria or fetal phenotype groups on ES diagnostic yield, heterogeneity persisted within and across many subgroups. This suggests that these factors do not fully account for the observed heterogeneity.

Funnel plot asymmetry in our review indicates potential publication bias, which might reflect a correlation between small sample sizes and elevated diagnosis rates. The studies in this review were predominantly selected for small cohorts with a genetic inclination towards a monogenic cause, often identified after expert genetic evaluation. This highly selective approach towards monogenic diseases could lead to a higher diagnostic yield. Furthermore, with 13 of the 21 studies conducted in China, the applicability of our findings may be influenced by specific demographic data.

Cases that had undergone whole genome sequencing (WGS) were considered in our screening of the literature, but because there are relatively few published studies of WGS testing in prenatal diagnosis of SKA fetuses, none were eligible for inclusion. In the future, with the wide application of WGS in prenatal diagnosis of SKA fetuses, more genetic causes may be found, and the detection rate of monogenic diseases in SKA fetuses will also increase. Most studies have evaluated the diagnostic value of prenatal ES for SKA fetuses only with a small sample size, and the low number of cases would make it difficult to find valuable information in our analysis. So in order to achieve a larger study cohort, we included different ES methods for detecting SKA fetuses, including gene panel testing, which may result in a slightly lower diagnostic yield. These are indeed a limitation of this study.

The final conclusions of this meta-analysis are indeed less valuable than we expected. At the beginning, we sought to find the difference in the detection rate of isolated and non-isolated short long bone cases and the correlation between the degree of shortening in isolated short long bone cases and the detection rate, but unfortunately, we did not find meaningful results. This is also a weakness of the analysis.

## 5 Conclusion

This meta-analysis concludes that genetic variation plays a significant role in the causation of fetal SKA, with the majority of cases attributed to single-gene variants. Consequently, it is essential to advocate for the prenatal diagnosis of SKA using ES in clinical settings. Trio ES should be performed first, especially in fetuses with pure skeletal dysplasia or classical skeletal dysplasia. In the future, with the wide application of WGS in the prenatal diagnosis of SKA fetuses, more genetic causes of SKA may be found, and the detection rate of SKA fetuses will also be improved.

## Data Availability

The original contributions presented in the study are included in the article/Supplementary Material, further inquiries can be directed to the corresponding authors.

## Supplementary material

SUPPLEMENTARY FIGURE S1 Forest plot of total detection rate.

SUPPLEMENTARY FIGURE S2 The proportion of various genes. In these 301 cases, a total of 76 genes across 304 types of variants were detected. The ten most common genes identified were *FGFR3, COL1A1, COL1A2, COL2A1, DYNC2H1, ALPL, FLNB, EBP, PPIB*, and *IFITM5*. Their respective frequencies were 31.3%, 14.5%, 10.2%, 8.9%, 3.6%, 2.3%, 1.3%, 1.3%, 1.3%, and 1.0%.

SUPPLEMENTARY FIGURE S3 Subgroup distribution of common variant genes.

SUPPLEMENTARY FIGURE S4 Funnel plot of total detection rate.

## References

[B1] Aggarwal S. Vineeth V. S. Das Bhowmik A. Tandon A. Kulkarni A. Narayanan D. L. (2019). Exome sequencing for perinatal phenotypes: the significance of deep phenotyping. Prenat. Diagn. 40 (2), 260–273. 10.1002/pd.5616 31742715

[B2] Augusciak-Duma A. Witecka J. Sieron A. L. Janeczko M. Pietrzyk J. J. Ochman K. (2018). Mutations in the COL1A1 and COL1A2 genes associated with osteogenesis imperfecta (OI) types I or III. Acta Biochim. Pol. 65 (1), 79–86. 10.18388/abp.2017_1612 29543922

[B3] Bai Y. Sun Y. Liu N. Wang L. Jiao Z. H. Hou Y. Q. (2022). Genetic analysis of 55 cases with fetal skeletal dysplasia. Orphanet J. Rare Dis. 17 (1), 410. 10.1186/s13023-022-02559-4 36352425 PMC9648031

[B4] Best S. Wou K. Vora N. Van der Veyver I. B. Wapner R. Chitty L. S. (2018). Promises, pitfalls and practicalities of prenatal whole exome sequencing. Prenat. Diagn 38 (1), 10–19. 10.1002/pd.5102 28654730 PMC5745303

[B5] Callaway J. L. Shaffer L. G. Chitty L. S. Rosenfeld J. A. Crolla J. A. (2013). The clinical utility of microarray technologies applied to prenatal cytogenetics in the presence of a normal conventional karyotype: a review of the literature. Prenat. Diagn 33 (12), 1119–1123. 10.1002/pd.4209 23983223 PMC4285999

[B6] Cao J. Chen A. E. Tian L. Y. Yan L. L. Li H. B. Zhou B. H. (2022). Application of whole exome sequencing in fetal cases with skeletal abnormalities. Heliyon 8 (7), e09819. 10.1016/j.heliyon.2022.e09819 35855989 PMC9287157

[B7] Chandler N. Best S. Hayward J. Faravelli F. Mansour S. Kivuva E. (2018). Rapid prenatal diagnosis using targeted exome sequencing: a cohort study to assess feasibility and potential impact on prenatal counseling and pregnancy management. Genet. Med. 20 (11), 1430–1437. 10.1038/gim.2018.30 29595812

[B8] Deden C. Neveling K. Zafeiropopoulou D. Gilissen C. Pfundt R. Rinne T. (2020). Rapid whole exome sequencing in pregnancies to identify the underlying genetic cause in fetuses with congenital anomalies detected by ultrasound imaging. Prenat. Diagn. 40 (8), 972–983. 10.1002/pd.5717 32333414 PMC7497059

[B9] de Wit M. C. Srebniak M. I. Govaerts L. C. Van Opstal D. Galjaard R. J. Go A. T. (2014). Additional value of prenatal genomic array testing in fetuses with isolated structural ultrasound abnormalities and a normal karyotype: a systematic review of the literature. Ultrasound Obstet. Gynecol. 43 (2), 139–146. 10.1002/uog.12575 23897843

[B10] Han J. Yang Y. D. He Y. Liu W. J. Zhen L. Pan M. (2020). Rapid prenatal diagnosis of skeletal dysplasia using medical trio exome sequencing: benefit for prenatal counseling and pregnancy management. Prenat. Diagn. 40 (5), 577–584. 10.1002/pd.5653 31994750

[B11] Huang Y. L. Liu C. Ding H. K. Wang Y. A. Yu L. H. Guo F. F. (2023). Exome sequencing in fetuses with short long bones detected by ultrasonography: a retrospective cohort study. Front. Genet. 14, 1032346. 10.3389/fgene.2023.1032346 36923788 PMC10010437

[B12] Hui A. S. Chau M. H. K. Chan Y. M. Cao Y. Kwan A. H. Zhu X. (2021). The role of chromosomal microarray analysis among fetuses with normal karyotype and single system anomaly or nonspecific sonographic findings. Acta Obstet. Gynecol. Scand. 100 (2), 235–243. 10.1111/aogs.14003 32981064

[B13] Jelin A. C. Blakemore K. Trebes S. Sagaser K. Forster K. R. Russo M. (2020). Molecular testing strategies in the evaluation of fetal skeletal dysplasia. J. Maternal-Fetal & Neonatal Med. 35 (14), 2788–2794. 10.1080/14767058.2020.1802715 PMC7858696 32752906

[B14] Krakow D. (2015). Skeletal dysplasias. Clin. Perinatology 42 (2), 301–319. 10.1016/j.clp.2015.03.003 PMC4456691 26042906

[B15] Kucinska-Chahwan A. Roszkowski T. Nowakowska B. Geremek M. Paczkowska M. Bijok J. (2022). Extended genetic testing in fetuses with sonographic skeletal system abnormalities. Ultrasound Obstetrics & Gynecol. 59 (5), 660–667. 10.1002/uog.23722 34198368

[B16] Liu Y. Wang L. Yang Y.-K. Liang Y. Zhang T.-J. Liang N. (2019). Prenatal diagnosis of fetal skeletal dysplasia using targeted next-generation sequencing: an analysis of 30 cases. Diagn. Pathol. 14 (1), 76. 10.1186/s13000-019-0853-x 31299979 PMC6626426

[B17] Lord J. McMullan D. J. Eberhardt R. Y. Rinck G. Hamilton S. J. Quinlan-Jones E. (2019). Prenatal exome sequencing analysis in fetal structural anomalies detected by ultrasonography (PAGE): a cohort study. Lancet 393 (10173), 747–757. 10.1016/S0140-6736(18)31940-8 30712880 PMC6386638

[B18] Marini J. C. Forlino A. Bachinger H. P. Bishop N. J. Byers P. H. Paepe A. (2017). Osteogenesis imperfecta. Nat. Rev. Dis. Prim. 3, 17052. 10.1038/nrdp.2017.52 28820180

[B19] Mellis R. Oprych K. Scotchman E. Hill M. Chitty L. S. (2022). Diagnostic yield of exome sequencing for prenatal diagnosis of fetal structural anomalies: a systematic review and meta-analysis. Prenat. Diagn. 42 (6), 662–685. 10.1002/pd.6115 35170059 PMC9325531

[B20] Milks K. S. Hill L. M. Hosseinzadeh K. (2017). Evaluating skeletal dysplasias on prenatal ultrasound: an emphasis on predicting lethality. Pediatr. Radiol. 47 (2), 134–145. 10.1007/s00247-016-3725-5 27904917

[B21] Moher D. Liberati A. Tetzlaff J. Altman D. G. PRISMA Group (2009). Preferred reporting items for systematic reviews and meta-analyses: the PRISMA statement. PLoS Med. 6 (7), e1000097. 10.1371/journal.pmed.1000097 19621072 PMC2707599

[B22] Ornitz D. M. Marie P. J. (2015). Fibroblast growth factor signaling in skeletal development and disease. Genes Dev. 29 (14), 1463–1486. 10.1101/gad.266551.115 26220993 PMC4526732

[B23] Page M. J. McKenzie J. E. Bossuyt P. M. Boutron I. Hoffmann T. C. Mulrow C. D. (2021). The PRISMA 2020 statement: an updated guideline for reporting systematic reviews. PLOS Med. 18 (3), e1003583. 10.1371/journal.pmed.1003583 33780438 PMC8007028

[B24] Peng Y. Yang S. T. Huang X. L. Pang J. L. Liu J. Hu J. C. (2021). Whole exome sequencing analysis in fetal skeletal dysplasia detected by ultrasonography: an analysis of 38 cases. Front. Genet. 12, 728544. 10.3389/fgene.2021.728544 34567078 PMC8461062

[B25] Petrovski S. Aggarwal V. Giordano J. L. Stosic M. Wou K. Bier L. (2019). Whole-exome sequencing in the evaluation of fetal structural anomalies: a prospective cohort study. Lancet 393 (10173), 758–767. 10.1016/S0140-6736(18)32042-7 30712878

[B26] Schramm T. Mommsen H. (2018). Fetal skeletal disorders. Ultraschall der Medizin - Eur. J. Ultrasound 39 (06), 610–634. 10.1055/a-0660-9417 30189431

[B27] Shi J. Ren M. Jia J. Tang M. Guo Y. Ni X. (2019). Genotype-phenotype association analysis reveals new pathogenic factors for osteogenesis imperfecta disease. Front. Pharmacol. 10, 1200. 10.3389/fphar.2019.01200 31680973 PMC6803541

[B28] Tang H. Zhang Q. Xiang J. J. Yin L. L. Wang J. Wang T. (2021). Whole exome sequencing aids the diagnosis of fetal skeletal dysplasia. Front. Genet. 12, 599863. 10.3389/fgene.2021.599863 33777089 PMC7987927

[B29] Tang J. Zhou C. L. Shi H. H. Mo Y. Y. Tan W. L. Sun T. L. (2020). Prenatal diagnosis of skeletal dysplasias using whole exome sequencing in China. Clin. Chim. Acta 507, 187–193. 10.1016/j.cca.2020.04.031 32360156

[B30] Tolusso L. K. Hazelton P. Wong B. Swarr D. T. (2021). Beyond diagnostic yield: prenatal exome sequencing results in maternal, neonatal, and familial clinical management changes. Genet. Med. 23 (5), 909–917. 10.1038/s41436-020-01067-9 33442022 PMC7804210

[B31] Tournis S. Dede A. D. (2018). Osteogenesis imperfecta - a clinical update. Metabolism 80, 27–37. 10.1016/j.metabol.2017.06.001 28625337

[B32] Tse K. Y. Surya I. U. Irwinda R. Leung K. Y. Ting Y. H. Cao Y. (2023). Diagnostic yield of exome sequencing in fetuses with sonographic features of skeletal dysplasias but normal karyotype or chromosomal microarray analysis: a systematic review. Genes 14 (6), 1203. 10.3390/genes14061203 37372383 PMC10298097

[B33] Unger S. Ferreira C. R. Mortier G. R. Ali H. Bertola D. R. Calder A. (2023). Nosology of genetic skeletal disorders: 2023 revision. Am. J. Med. Genet. Part A 191 (5), 1164–1209. 10.1002/ajmg.a.63132 36779427 PMC10081954

[B34] Vora N. L. Powell B. Brandt A. Strande N. Hardisty E. Gilmore K. (2017). Prenatal exome sequencing in anomalous fetuses: new opportunities and challenges. Genet. Med. 19 (11), 1207–1216. 10.1038/gim.2017.33 28518170 PMC5675748

[B35] Waller D. K. Correa A. Vo T. M. Wang Y. Hobbs C. Langlois P. H. (2008). The population‐based prevalence of achondroplasia and thanatophoric dysplasia in selected regions of the US. Am. J. Med. Genet. Part A 146A (18), 2385–2389. 10.1002/ajmg.a.32485 18698630 PMC6034636

[B36] Wapner R. J. Martin C. L. Levy B. Ballif B. C. Eng C. M. Zachary J. M. (2012). Chromosomal microarray versus karyotyping for prenatal diagnosis. N. Engl. J. Med. 367 (23), 2175–2184. 10.1056/NEJMoa1203382 23215555 PMC3549418

[B37] Yadava S. M. Ashkinadze E. (2018). Whole exome sequencing for prenatal diagnosis in cases with fetal anomalies: criteria to improve diagnostic yield. J. Genet. Couns. 28 (2), 251–255. 10.1002/jgc4.1045 30629328

[B38] Yang K. Shen M. Yan Y. S. Tan Y. Zhang J. Wu J. (2019). Genetic analysis in fetal skeletal dysplasias by trio whole-exome sequencing. Biomed Res. Int. 2019, 2492590. 10.1155/2019/2492590 31218223 PMC6537022

[B39] Yang Y. Wang M. Wang H. (2022). Prenatal trio-based whole exome sequencing in fetuses with abnormalities of the skeletal system. Mol. Genet. Genomics 297 (4), 1017–1026. 10.1007/s00438-022-01899-x 35583673

[B40] Zhang B. Zhang Y. Wu N. Li J. Liu H. Wang J. (2020). Integrated analysis of COL2A1 variant data and classification of type II collagenopathies. Clin. Genet. 97 (3), 383–395. 10.1111/cge.13680 31758797

[B41] Zhang L. Pan L. J. Teng Y. L. Liang D. S. Li Z. Wu L. Q. (2021a). Molecular diagnosis for 55 fetuses with skeletal dysplasias by whole-exome sequencing: a retrospective cohort study. Clin. Genet. 100 (2), 219–226. 10.1111/cge.13976 33942288

[B42] Zhang X. Y. Ren Y. Song R. Wang L. X. Xu H. Xie X. X. (2021b). Combined exome sequencing and deep phenotyping in highly selected fetuses with skeletal dysplasia during the first and second trimesters improves diagnostic yield. Prenat. Diagn. 41 (11), 1401–1413. 10.1002/pd.5974 34091931

[B43] Zhou X. Chandler N. Deng L. Zhou J. Yuan M. Sun L. (2018). Prenatal diagnosis of skeletal dysplasias using a targeted skeletal gene panel. Prenat. Diagn. 38 (9), 692–699. 10.1002/pd.5298 29907962

[B44] Zhytnik L. Maasalu K. Reimann E. Prans E. Koks S. Martson A. (2017). Mutational analysis of COL1A1 and COL1A2 genes among Estonian osteogenesis imperfecta patients. Hum. Genomics 11 (1), 19. 10.1186/s40246-017-0115-5 28810924 PMC5558703

