# Supplementary material for: Exome sequencing and prenatal skeletal abnormalities: comprehensive review and meta-analysis and way forward
Source: Front Genet. 2025 Jun 11;16:1502538. doi: 10.3389/fgene.2025.1502538 (PMC12188644; doi:10.3389/fgene.2025.1502538)
Supplement: Supplementary file 1 [file Table1.doc]

**STable 1.** Quality evaluation of the included literature according to the 11 items recommended by AHRQ

| **Studies** | **Cohort studies** | **Rule out chromosomal abnormalities** | **Time of Detection** | **SKA fetuses** | **Influence of subjective factors** | **Validation of parents** | **Grounds for exclusion** | **Assessing confounding factors** | **Missing data or not** | **Complete data** | **Follow-up** |
| --- | --- | --- | --- | --- | --- | --- | --- | --- | --- | --- | --- |
| Cao et al., 2022 | Yes | Yes | Yes | Yes | Not clear | Yes | Yes | Not clear | Not clear | Yes | No |
| Zhang et al., 2021b | Yes | Yes | Yes | Yes | Not clear | Yes | Yes | Not clear | Not clear | Yes | Yes |
| Zhang et al., 2021a | Yes | Yes | Yes | Yes | Not clear | Yes | Yes | Not clear | Not clear | Yes | No |
| Yang et al., 2019 | Yes | Yes | Yes | Yes | Not clear | Yes | Yes | Not clear | Not clear | Yes | Yes |
| Yang et al., 2022 | Yes | Yes | Yes | Yes | Not clear | Yes | Yes | Not clear | Not clear | Yes | No |
| Huang et al., 2023 | Yes | Yes | Yes | Yes | Not clear | Yes | Yes | Not clear | Not clear | Yes | Yes |
| Kucinska-Chahwan et al., 2022 | Yes | Yes | Yes | Yes | Not clear | Yes | Yes | Not clear | Not clear | Yes | No |
| Bai et al., 2022 | Yes | Yes | Yes | Yes | Not clear | Yes | Yes | Not clear | Not clear | Yes | Yes |
| Peng et al., 2021 | Yes | Yes | Yes | Yes | Not clear | Yes | Yes | Not clear | Not clear | Yes | Yes |
| Tang et al., 2021 | Yes | Yes | Yes | Yes | Not clear | Yes | Yes | Not clear | Not clear | Yes | No |
| Deden et al., 2020 | Yes | Yes | Yes | Yes | Not clear | Yes | Yes | Not clear | Not clear | Yes | Yes |
| Chandler et al., 2018 | Yes | Yes | Yes | Yes | Not clear | Yes | Yes | Not clear | Not clear | Yes | Yes |
| Han et al., 2020 | Yes | Yes | Yes | Yes | Not clear | Yes | Yes | Not clear | Not clear | Yes | Yes |
| Tang et al., 2020 | Yes | Yes | Yes | Yes | Not clear | Yes | Yes | Not clear | Not clear | Yes | Yes |
| Liu et al., 2019 | Yes | Yes | Yes | Yes | Not clear | Yes | Yes | Not clear | Not clear | Yes | No |
| Tolusso et al., 2021 | Yes | Yes | Yes | Yes | Not clear | Yes | Yes | Not clear | Not clear | Yes | Yes |
| Zhou et al., 2018 | Yes | Yes | Yes | Yes | Not clear | Yes | Yes | Not clear | Not clear | Yes | Yes |
| Aggarwal et al., 2019 | Yes | Yes | Yes | Yes | Not clear | No | Yes | Not clear | Not clear | Yes | Yes |
| Jelin et al., 2020 | Yes | Yes | Yes | Yes | Not clear | Yes | Yes | Not clear | Not clear | Yes | Yes |
| Yadava and Ashkinadze, 2018 | Yes | Yes | Yes | Yes | Not clear | Yes | Yes | Not clear | Not clear | Yes | Yes |
| Vora et al., 2017 | Yes | Yes | Yes | Yes | Not clear | Yes | Yes | Not clear | Not clear | Yes | Yes |
