# Supplementary material for: Exome sequencing and prenatal skeletal abnormalities: comprehensive review and meta-analysis and way forward
Source: Front Genet. 2025 Jun 11;16:1502538. doi: 10.3389/fgene.2025.1502538 (PMC12188644; doi:10.3389/fgene.2025.1502538)
Supplement: Supplementary file 3 [file Table2.doc]

**STable 2.** Comparative analysis of the detection rates of monogenic abnormalities in different subgroups of SKA fetuses

| **Group** | **Detection rate of monogenic abnormalities** | **p Value** |
| --- | --- | --- |
| SKA |  |  |
| Isolated SKA vs SKA combined with other abnormalities | 68.6%(203/296) vs 54.4%(98/180) | 0.07 |
| Short long bones |  |  |
| Isolated short long bones vs Non-isolated short long bones | 61.9%（52/84）vs 68.5%(198/289) | 0.35 |
| Short long bones only combined with other skeletal abnormalities vs Short long bones combined with both other skeletal abnormalities and non-skeletal abnormalities | 79.1%（129/163）vs 65.2%（45/69） | 0.04 |
| <-2SD vs <-4SD | 22.2%(2/9) vs 50.0%(4/8) | 0.66 |
| Isolated curvature abnormalities of long bones vs Long bone curvature abnormalities associated with short long bones only | 100.0%(3/3) vs 82.6%(38/46) | 0.59 |
| Long bone curvature abnormalities associated with short long bones only vs Short long bones combined with both other skeletal abnormalities and non-skeletal abnormalities | 82.6%(38/46) vs 65.2%（45/69） | 0.04 |
| Subgroup phenotype |  |  |
| Suspected fractures or angulated long bones vs Absence of long bones | 78.1%(25/32) vs 31.3%(15/48) | <0.00001 |
| Suspected fractures or angulated long bones vs Abnormal joint posture | 78.1%(25/32) vs 48.5%(33/68) | 0.01 |
| Suspected fractures or angulated long bones vs Spinal abnormalities | 78.1%(25/32) vs 55.3%(21/38) | 0.02 |
| Abnormal ossification vs Absence of long bones | 85.0%(17/20) vs 31.3%(15/48) | <0.00001 |
| Abnormal ossification vs Abnormal joint posture | 85.0%(17/20) vs 48.5%(33/68) | 0.004 |
| Abnormal ossification vs Facial abnormalities | 85.0%(17/20) vs 55.9%(19/34) | 0.02 |
| Abnormal ossification vs Spinal abnormalities | 85.0%(17/20) vs 55.3%(21/38) | 0.009 |
| Abnormal ossification vs Multiple system anomaly | 85.0%(17/20) vs 54.9%(45/82) | 0.03 |
| Absence of long bones vs Abnormal joint posture | 31.3%(15/48) vs 48.5%(33/68) | 0.04 |
| Absence of long bones vs Abnormalities of the skull | 31.3%(15/48) vs 77.8%(35/45) | <0.0001 |
| Absence of long bones vs Facial abnormalities | 31.3%(15/48) vs 55.9%(19/34) | 0.02 |
| Absence of long bones vs Small breasts | 31.3%(15/48) vs 81.5%(66/81) | <0.00001 |
| Absence of long bones vs Multiple system anomaly | 31.3%(15/48) vs 54.9%(45/82) | 0.004 |
| Abnormal joint posture vs Abnormalities of the skull | 48.5%(33/68) vs 77.8%(35/45) | 0.03 |
| Abnormal joint posture vs Small breasts | 48.5%(33/68) vs 81.5%(66/81) | <0.0001 |
| Abnormalities of the skull vs Spinal abnormalities | 77.8%(35/45) vs 55.3%(21/38) | 0.004 |
| Facial abnormalities vs Small breasts | 55.9%(19/34) vs 81.5%(66/81) | 0.002 |
| Small breasts vs Spinal abnormalities | 81.5%(66/81) vs 55.3%(21/38) | 0.0009 |
| Small breasts vs Multiple system anomaly | 81.5%(66/81) vs 54.9%(45/82) | 0.002 |

Additional Note:

Among the 476 SD fetuses included in the study, the detection rate of isolated SD fetuses was 68.6%(203/296), and the detection rate of non-isolated SD fetuses was 54.4%(98/180), but the two groups did not achieve statistical significance (p=0.07). Our study revealed that the detection rate of fetuses with isolated short long bones (52/84, 61.9%) was lower compared to those with non-isolated short long bones (198/289, 68.5%). However, this difference was not statistically significant (p=0.35). The detection rate of short long bone only combined with other skeletal abnormalities (129/163, 79.1%) was significantly higher than that of short long bone combined with both other skeletal abnormalities and non-skeletal abnormalities (65.2%, 45/69) and p value was 0.04. When comparing fetuses with short bones less than –2SD to those with short bones below –4SD, the latter were more likely to be detected genetic abnormality with ES (22.2% for –2SD versus 50.0% for –4SD), but this difference did not reach statistical significance (p=0.66). However, there was no significant difference in the detection rate of ES between the groups of isolated long bone curvature abnormality and long bone curvature abnormality combined with short long bones only (P=0.59). There was no statistically significant difference in the detection rate of the three subgroups of abnormal curvature. However, pairwise comparison between the subgroup of abnormal curvature and the short-long bone subgroup showed that the detection rate of abnormal curvature of long bone only combined with short-long bone (38/46, 82.6%) was higher than that of short-long bone combined with other skeletal abnormalities and non-skeletal abnormalities (45/69, 65.2%), and there was statistically significant (p=0.04).

Pairwise comparisons between subgroups showed that the detection rate of suspected fractures or angulated long bones was significantly higher than that of absence of long bones, abnormal joint posture, and spinal abnormalities (p<0.00001, p=0.01, p=0.02, respectively). The detection rate of abnormal ossification was significantly higher than that of absence of long bones, abnormal joint posture, facial abnormalities, spinal abnormalities, and multiple system anomaly (p<0.00001, p=0.004, p=0.02, p=0.009, p=0.03, respectively). The detection rate of absence of long bones was significantly lower than that of abnormal joint posture, abnormalities of the skull, facial abnormalities, small breasts, and multiple system anomaly (p=0.04, p<0.0001, p=0.02, p<0.00001, p=0.004, respectively). The detection rate of abnormal joint posture was significantly lower than that of abnormalities of the skull and small breasts subgroups (p=0.03, p<0.0001, respectively). The detection rate of abnormalities of the skull was significantly higher than that of spine abnormalities (p=0.004). The detection rate of facial abnormalities was significantly lower than that of small breasts (p=0.002). The detection rate of small breasts was significantly higher than that of spinal abnormalities and multiple system anomaly (p=0.0009, p=0.002, respectively). There was no statistical significance among the other subgroups.
